# Development of Levo-Lansoprazole Chiral Molecularly Imprinted Polymer Sensor Based on the Polylysine–Phenylalanine Complex Framework Conformational Separation

**DOI:** 10.3390/bios13050509

**Published:** 2023-04-28

**Authors:** Lianming Zhang, Zian Wang, Dan Li, Yali Yuan, Huixiang Ouyang, Jianping Li

**Affiliations:** 1 College of Chemistry and Bioengineering, Guilin University of Technology, Guilin 541004, China; 2 College of Chemistry & Environment Engineering, Baise University, Baise 533000, China

**Keywords:** molecularly imprinted, complex framework, selectivity, enantiomer, levo-lansoprazole

## Abstract

The efficacies and toxicities of chiral drug enantiomers are often dissimilar, necessitating chiral recognition methods. Herein, a polylysine–phenylalanine complex framework was used to prepare molecularly imprinted polymers (MIPs) as sensors with enhanced specific recognition capabilities for levo-lansoprazole. The properties of the MIP sensor were investigated using Fourier-transform infrared spectroscopy and electrochemical methods. The optimal sensor performance was achieved by applying self-assembly times of 30.0 and 25.0 min for the complex framework and levo-lansoprazole, respectively, eight electropolymerization cycles with *o*-phenylenediamine as the functional monomer, an elution time of 5.0 min using an ethanol/acetic acid/H_2_O mixture (2/3/8, *V*/*V*/*V*) as the eluent, and a rebound time of 10.0 min. A linear relationship was observed between the sensor response intensity (Δ*I*) and logarithm of the levo-lansoprazole concentration (l-g *C*) in the range of 1.0 × 10^−13^–3.0 × 10^−11^ mol/L. Compared with a conventional MIP sensor, the proposed sensor showed more efficient enantiomeric recognition, with high selectivity and specificity for levo-lansoprazole. The sensor was successfully applied to levo-lansoprazole detection in enteric-coated lansoprazole tablets, thus demonstrating its suitability for practical applications.

## 1. Introduction

Chirality [1] is a widely observed phenomenon in nature and an inherent feature of the molecular and macromolecular components of organisms, including tissues, cells, proteins, nucleic acids, and polysaccharides. Owing to its universality and significance, substantial research efforts have been directed towards studying various aspects of chirality, and the application of chiral drugs is an important trend in the field of medicine. From a pharmacological perspective, generally, only one enantiomer of chiral drugs is medicinally important, whereas the other is not effective and may even exhibit toxic side effects [2]. Therefore, analytical methods that differentiate chiral drug enantiomers are important [3]. Based on the regulations established by the U.S. Food and Drug Administration (FDA) in 1992 [4], for drugs containing chiral centers, the physical and chemical properties of each enantiomer must be determined. If the pharmacokinetics of the enantiomers are dissimilar, the linear relationship between their respective doses and any interaction effects between the metabolites and the drug should be established. The toxicity and side effects of each enantiomer must be determined to prevent possible harmful effects within the human body. The chiral drug regulations of the FDA, as well as the corresponding European regulations, promote the development of single-enantiomer drugs. Therefore, research on the detection of chiral drug enantiomers can have far-reaching implications for clinical applications and improving rational drug use.

Lansoprazole [5] is a proton-pump inhibitor that is mainly used for the treatment of duodenal ulcers, reflux esophagitis, and gastric ulcers. Furthermore, it has been recognized as a new type of drug for the treatment of breast tumors [6]. Owing to the presence of a lone pair of electrons on the S atom in its structure, lansoprazole exists as a pair of chiral enantiomers. Although both l- and d-isomers exhibit therapeutic effects, dexlansoprazole is more effective than levo-lansoprazole [7]. Currently, high-performance liquid chromatography (HPLC) [8] and HPLC–mass spectrometry (HPLC-MS) are predominantly used for the detection of levo-lansoprazole [9,10]. However, these methods require expensive instrumentation, lengthy operation times, and cannot be used for real-time analyses. Therefore, the development of rapid chiral separation methods featuring excellent selectivity and high sensitivity is essential.

Molecularly imprinted polymer (MIP) sensors [11,12,13] prepared using molecular imprinting technology (MIT) [14] are well-known for their advantageous features, including structural predictability and recognition specificity. The molecular imprinting technique involves the synthesis of a polymer in the presence of a target molecule as a template, resulting in the formation of molecular recognition sites in the polymer. During polymerization, the complementary interactions between the functional monomer and target molecule are maintained; this spatial arrangement can be further stabilized via polymer crosslinking. Therefore, the generated MIP sensors can selectively recognize the target analyte via the template-derived sites [15,16]. MIP sensors have been applied to chiral separation for many years, and their use in the field of medicine continues to increase [17,18,19,20]. However, in practice, conventional MIP sensors display poor separation and recognition capabilities, often recognizing substances that are structurally similar to the target molecule; moreover, their stability is relatively low [21]. To address these shortcomings, a rapid detection method that allows for highly efficient separation and recognition is required.

**Figure 1:**
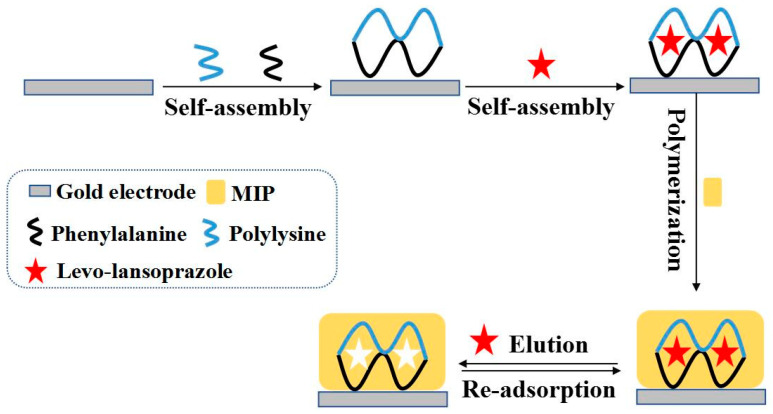
Schematic diagram of the MIP sensor for levo-lansoprazole immobilized by the polylysine–phenylalanine complex framework.

In this study, to improve sensor selectivity, a polylysine–phenylalanine complex framework was used to prepare the MIPs. Owing to its highly ordered three-dimensional (3D) structure, the framework anchored the spatial conformation of the target molecule while simultaneously improving the structural fineness of the imprinted pores to increase the number of recognition sites. Using levo-lansoprazole as the target molecule and *o*-phenylenediamine as the functional monomer, and MIP capable of highly selective molecular conformation recognition was constructed via electropolymerization. The subsequent elution of levo-lansoprazole delivered a chiral MIP sensor bearing complementary imprinted pores that matched the spatial conformation, size, and structure of levo-lansoprazole. Owing to these recognition sites, the sensor exhibited high selectivity and specificity for levo-lansoprazole upon re-adsorption. The recognition mechanism is illustrated in Figure 1.

## 2. Materials and Methods

### 2.1. Materials and Instruments

Polylysine was purchased from Wuhan Dongkangyuan Technology Co., Ltd., Wuhan, China (www.dkybpc.com, accessed on 24 February 2021), while *L-β*-Phenylalanine was purchased from Shanghai Aladdin Biochemical Technology Co., Ltd., Shanghai, China (www.aladdin-e.com, accessed on 21 March 2021). Omeprazole, rabeprazole, lansoprazole sulfone, lansoprazole sulphide, lansoprazole *N*-ethylene oxide, and levo-lansoprazole were purchased from Hubei Guangao Biotechnology Co., Ltd., Tianmen, China (www.guangaobio.comt, accessed on 17 July 2021). Dextro-lansoprazole was obtained from Shanghai Bide Pharmatech, Ltd., Shanghai, China (www.bidepharmatech.com, accessed on 13 April 2021), while lansoprazole enteric-coated lansoprazole tablets were purchased from Jiangsu Kanion Pharmaceutical Co., Ltd., Lianyungang, China (www.kanion.com, accessed on 11 February 2022). All reagents were of analytical grade. All aqueous solutions were prepared using doubly distilled water.

Cyclic voltammetry (CV) and differential pulse voltammetry (DPV) measurements were performed using a CHI600D electrochemical workstation (Shanghai Chenhua Instrument Co., Ltd., Shanghai, China; www.chinstr.com, accessed on 11 July 2018). A three-electrode system was used comprising Ag/AgCl (saturated KCl) as the reference electrode, a platinum disc as the counter electrode, and a MIP-modified gold electrode as the working electrode. Fourier-transform infrared (FTIR) spectra were collected using a Nicolet iS10 FTIR spectrometer (Thermo Fisher Technology Co., Ltd., Waltham, MA, USA; www.thermofisher.com, accessed on 19 May 2019).

### 2.2. Preparation of MIP and Non-MIP (nMIP) Sensors

A gold electrode was polished on suede using 1.0, 0.3, and 0.05 μm alumina polishing powder until a mirror surface finish was achieved. The polished electrode was ultrasonically cleaned successively in 50% nitric acid, absolute ethanol, and doubly distilled water for 5.0 min in each liquid. The cleaned gold electrode was immersed in 5.0 mL of a polylysine–phenylalanine complex framework solution (1.0 × 10^−4^ mol/L), and self-assembly was allowed to proceed for 30.0 min. Subsequently, the electrode was immersed in 5.0 mL of levo-lansoprazole solution (1.0 × 10^−4^ mol/L), and self-assembly was allowed to proceed for 25.0 min. Finally, the electrode was placed in a 1.0 × 10^−4^ mol/L *o*-phenylenediamine solution in 0.01 mol/L phosphate buffer (pH 7.4), and 8 electropolymerization cycles were performed using CV. The scanning range was −0.2 to +0.6 V, and the scanning rate was 50 mV/s. The prepared MIP membrane-modified electrode was immersed in an ethanol/acetic acid/H_2_O mixture (2/3/8, *V*/*V*/*V*) and stirred for 5.0 min to elute levo-lansoprazole, thereby affording the MIP sensor. As a control to verify the specific recognition ability of the MIP and determine whether levo-lansoprazole was adsorbed onto the sensor via physical adsorption, a nMIP sensor was prepared using the same method, except that the target molecule self-assembly step was omitted. 

A conventional MIP sensor was prepared via electropolymerization using a solution of 1.0 × 10^−4^ mol/L levo-lansoprazole and 1.0 × 10^−4^ mol/L *o*-phenylenediamine in 0.01 mol/L phosphate buffer (pH 7.4). Eight polymerization cycles were performed using CV with a scanning range of −0.2 to +0.6 V at a scanning rate of 50 mV/s. The electropolymerized electrode was then immersed in an ethanol/acetic acid/H_2_O mixture (2/3/8, *V*/*V*/*V*) and stirred for 5.0 min to elute levo-lansoprazole, thereby affording the conventional MIP sensor.

### 2.3. Electrochemical Measurements

Using K_3_[Fe(CN)_6_] as a probe molecule (5.0 × 10^−3^ mol/L K_3_[Fe(CN)_6_] solution containing 0.1 mol/L KCl), the performance of the MIP was evaluated through electrochemical methods. The CV measurements were conducted using scanning potential ranging from −0.2 to +0.6 V and a scan rate of 50 mV/s. For the DPV measurements, the sweep potential range, sweep rate, and amplitude were +0.6 to −0.2 V, 50 mV/s, and 50 mV, respectively. Electrochemical impedance spectroscopy (EIS) was carried out by applying a potential of 0.19 V, an alternating current of 10 mV, with a frequency range of 100 mHz–100 kHz.

### 2.4. Determination of Levo-Lansoprazole in Real Samples

The levo-lansoprazole content of commercially available enteric-coated lansoprazole tablets was determined as follows. Briefly, five enteric-coated lansoprazole tablets were ground into powder using a mortar and pestle. Then, 0.0250 g of powder were dissolved in 0.01 mol/L phosphate buffer (pH 7.4) and transferred to a 50.0 mL volumetric flask, which was made up to volume using the phosphate buffer. Furthermore, fresh human serum samples, which were collected from patients at the affiliated hospital of Guilin University of Technology and adequately diluted, were subjected to the standard addition method for the detection of levo-lansoprazole to evaluate the performance of the sensor. The re-adsorption of levo-lansoprazole onto the prepared MIP sensor was performed using the prepared sample solutions, and the corresponding DPV responses were recorded.

## 3. Results and Discussion

### 3.1. Electrochemical Performance of MIP Sensors

**Figure 2:**
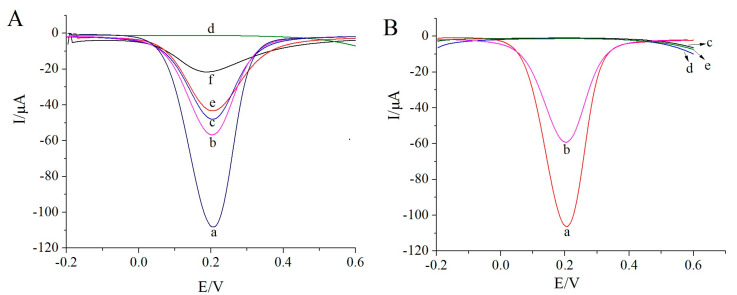
(**A**) DPV response of the MIP sensor. a: bare gold electrode, b: electrode modified with the polylysine–phenylalanine complex framework, c: 1.0 × 10^−12^ mol/L levo-lansoprazole self-assembled on the modified electrode, d: MIP-modified electrode, e: MIP-modified electrode after levo-lansoprazole elution, and f: MIP-modified electrode after the re-adsorption of 1.0 × 10^−12^ mol/L levo-lansoprazole. (**B**) DPV response of the nMIP sensor. a: a bare gold electrode, b: electrode modified with the polylysine–phenylalanine complex framework, c: nMIP-modified electrode, d: nMIP-modified electrode after levo-lansoprazole elution, and e: nMIP-modified electrode after the re-adsorption of 1.0 × 10^−12^ mol/L levo-lansoprazole.

To verify the feasibility of the proposed sensor, DPV was employed to characterize the electrochemical performance of the MIP membrane in response to K_3_[Fe(CN)_6_] as a probe molecule. As illustrated in Figure 2A, the reduction peak current of the electrode modified with the polylysine–phenylalanine complex framework (curve b) was significantly lower than that of the bare gold electrode (curve a). The distinction occurred, because the complex framework hindered electron transfer to a certain extent. Following levo-lansoprazole self-assembly on the electrode, the response signal decreased further (curve c). Levo-lansoprazole contains three F atoms, which can form hydrogen bonds with the H atoms of the –NH groups in the polylysine–phenylalanine framework, thereby immobilizing levo-lansoprazole onto the framework and further hindering electron transfer. The subsequent electropolymerization formed a dense MIP membrane with poor conductivity on the surface of the modified electrode. This membrane significantly decreased the electron transfer efficiency, leading to a rapid decrease in the signal intensity (curve d). Following levo-lansoprazole elution, imprinted pores, which were now available to participate in electron transfer, were obtained; therefore, the signal intensity increased significantly (curve e). When the sensor was placed in a levo-lansoprazole solution, rebinding of the target molecule in the imprinted cavities blocked the electron transfer channels again, resulting in a decrease in the signal intensity (curve f). These observations indicated that the prepared sensor was capable of identifying levo-lansoprazole.

To exclude the possibility of nonspecific adsorption, a nMIP sensor was also prepared and tested. As shown in Figure 2B, the response signal intensity of the electrode modified with the polylysine–phenylalanine complex framework (curve b) was significantly lower than that of the bare gold electrode (curve a). Furthermore, the signal for the nMIP-modified electrode was considerably lower than both abovementioned responses (curve c). However, following elution, the signal intensity (curve d) was consistent with that measured after electropolymerization. Moreover, after the rebinding of levo-lansoprazole, the signal intensity was similar to that after elution (curve e). Since levo-lansoprazole was absent during nMIP polymerization, molecularly imprinted pores did not form after elution, and thus, no electron transfer channels formed.

### 3.2. Impedance Response of the MIP Membrane under Different Conditions

**Figure 3:**
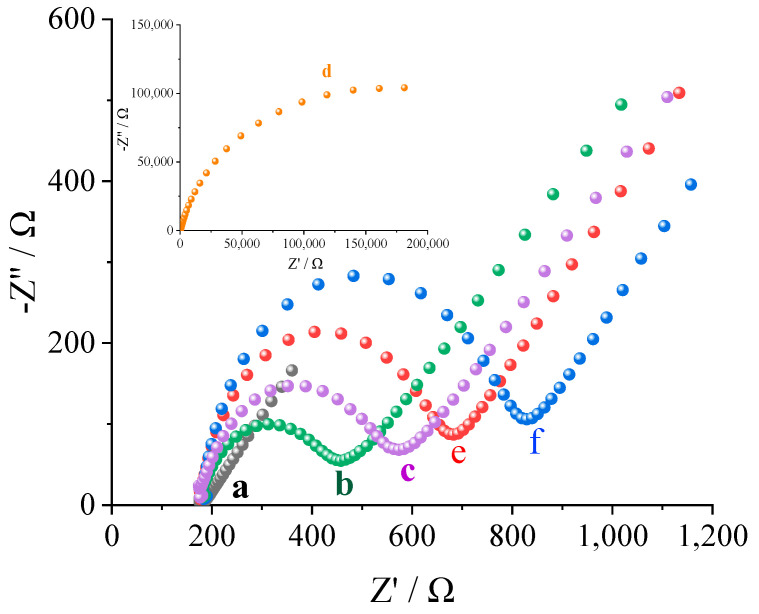
EIS data for the MIP sensor. a: a bare gold electrode, b: electrode modified with the polylysine–phenylalanine complex framework, c: 1.0 × 10^−12^ mol/L levo-lansoprazole self-assembled on the modified electrode, d: MIP-modified electrode, e: MIP-modified electrode after levo-lansoprazole elution, and f: MIP-modified electrode after the re-adsorption of 1.0 × 10^−12^ mol/L levo-lansoprazole.

EIS was used to evaluate the impedance response of the sensor under various conditions and characterize the MIP membrane on the surface of the gold electrode. As shown in Figure 3, the bare gold electrode exhibited the best conductivity under all conditions, displaying the lowest resistance (curve a, 183.09 Ω). When the electrode surface was modified with the polylysine–phenylalanine complex framework, electron transfer was hindered and the current flow was reduced, resulting in increased resistance (curve b, 457.67 Ω). After levo-lansoprazole was immobilized in the complex framework through hydrogen bonding, electron transfer was impeded and the resistance increased further (curve c, 573.53 Ω). After electropolymerization, the electrode surface was uniformly covered by a dense MIP membrane with poor conductivity, which led to a dramatic increase in the resistance (curve d, 181,811.76 Ω). However, once the target molecule was eluted, imprinted pores that could transfer electrons were formed, thereby lowering the resistance (curve e, 682.59 Ω). However, levo-lansoprazole re-adsorption in the imprinted pores blocked electron transfer, thereby increasing the resistance again (curve f, 829.06 Ω). These studies show that MIP membranes can recognize levo-lansoprazole.

### 3.3. FTIR Characterization

**Figure 4:**
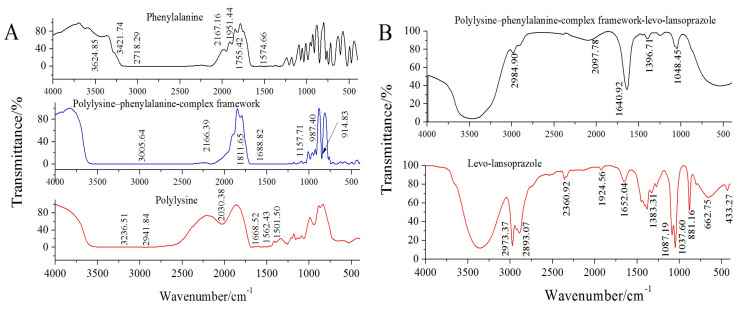
FTIR spectra of the polylysine−phenylalanine complex framework and levo−lansoprazole. (**A**): Phenylalanine, Polylysine−phenylalanine−complex framework, Polylysine; (**B**): Polylysine−phenylalanine−complex framework−levo−lansoprazole, Levo−lansoprazole.

FTIR characterization was used to verify the structures and bonding conditions of the polylysine–phenylalanine-complex framework and target molecule. As shown in Figure 4A, in the spectrum of polylysine, a stretching vibration peak of -C=O appeared at 1688.52 cm^−1^, and a strong absorption peak appeared at 1580–1520 cm^−1^. In phenylalanine, a carboxyl absorption peak appeared at 1574.66 cm^−1^, a carboxylic anion absorption peak appeared at 1755.42 cm^−1^, and a stretching absorption peak of N-H appeared at 2600–3100 cm^−1^. When polylysine was combined with phenylalanine, the stretching vibration of N-H was strengthened, and the characteristic broad peak at 3005.64 cm^−1^ was attributable to the carboxyl group. The above results proved that the polylysine–phenylalanine complex framework was constructed successfully [22,23].

To verify the assembly mechanism of the polylysine–phenylalanine complex framework and the bonding mode between the framework and levo-lansoprazole, FTIR characterization was performed. As shown in Figure 4B, levo-lansoprazole exhibited absorption peaks at 1037.60 and 1087.19 cm^−1^, which were attributed to the stretching vibrations of the sulfoxide and trifluoromethyl groups, respectively. The absorption peak at 1652.04 cm^−1^ was assigned to the stretching vibration of benzene. Based on the position of the peak at 881.16 cm^–1^, the benzene ring contained four adjacent H atoms (i.e., ortho-disubstituted benzene). The strong absorption peaks at 2973.37 and 2893.07 cm^−1^ corresponded to the stretching vibrations of the methyl and methylene groups on the pyridine ring, respectively. The absorption peak at 1383.31 cm^−1^ was attributed to the flexural vibration of the methyl group. When levo-lansoprazole was immobilized in the polylysine–phenylalanine complex framework, the trifluoromethyl absorption peak at 1087.19 cm^−1^ disappeared, indicating that levo-lansoprazole interacted with the framework through hydrogen bonds.

### 3.4. Optimization of Experimental Conditions

To optimize the prepared sensor, the following parameters were considered: self-assembly time for the complex framework, self-assembly time for the target molecule, number of polymerization cycles, eluent, elution time, and rebinding time. The corresponding data are provided in the electronic Supplementary Materials (Figures S1–S5). The best results were obtained using a self-assembly time of 30.0 min for the complex framework, a self-assembly time of 25.0 min for the target molecule, eight polymerization cycles, a mixture of ethanol/acetic acid/H_2_O (2/3/8, *V*/*V*/*V*) as the eluent with an elution time of 5.0 min, and a rebinding time of 10.0 min.

### 3.5. Working Curve

**Figure 5:**
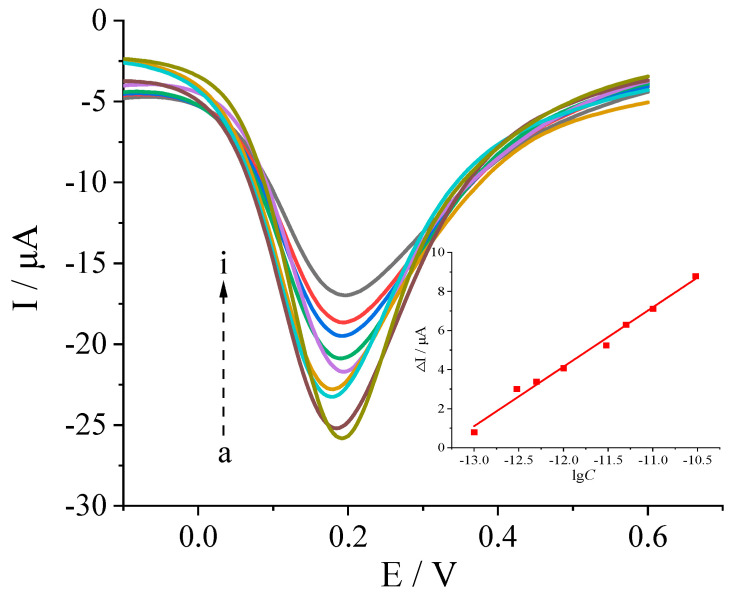
DPV response of the MIP sensor to different concentrations of levo−lansoprazole. a–i: 0, 1.0 × 10^−13^, 3.0 × 10^−13^, 5.0 × 10^−13^, 1.0 × 10^−12^, 3.0 × 10^−12^, 5.0 × 10^−12^, 1.0 × 10^−11^, and 3.0 × 10^−11^ mol/L.

Under the optimal conditions, the response of the sensor to varying concentrations of the target molecule levo-lansoprazole was investigated. As shown in Figure 5, the response signal intensity (Δ*I*) of the sensor decreased as the logarithm of the levo-lansoprazole concentration (lg *C*) increased, with a relatively good positive correlation observed in the range 1.0 × 10^−13^ to 3.0 × 10^−11^ mol/L. The corresponding linear equation is Δ*I* (μA) = 3.03lg *C* (mol/L) + 40.42 (r = 0.9947). Furthermore, the detection limit was determined to be 4.79 × 10^−14^ mol/L (DL = KSb/a, K = 3).

### 3.6. Chiral Separation Effcienct

**Figure 6:**
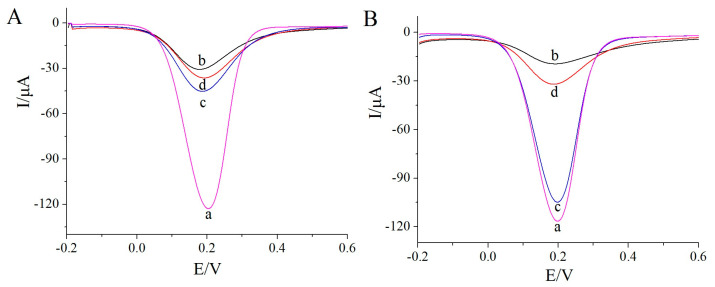
Sensitivities of (**A**) the conventional MIP sensor and (**B**) MIP sensor fabricated using the polylysine–phenylalanine complex framework.

To study the chiral separation efficiency of the MIP sensor fabricated using the polylysine–phenylalanine complex framework, its performance was compared with that of a conventional MIP sensor for the recognition of levo-lansoprazole, dexlansoprazole, and their racemate at a concentration of 1.0 × 10^−12^ mol/L. The results for the conventional MIP sensor are shown in Figure 6A, where the DPV response of the bare gold electrode is given by curve a. The conventional MIP sensor exhibited responses for both levo-lansoprazole (curve b) and dexlansoprazole (curve c); the response signal intensity for the racemate (curve d) was greater than half that of the levo-lansoprazole response at the same concentration. Since the imprinted pores formed by conventional methods are unstable and have a lower precision than those formed using the polylysine–phenylalanine complex framework, the presence of enantiomers can interfere with the recognition process to a greater extent. 

The recognition efficiency of the MIP sensor prepared using the polylysine–phenylalanine complex is shown in Figure 6B. This sensor showed a relatively strong response signal intensity for levo-lansoprazole, whereas the signal intensity for dexlansoprazole was only 3.33% that of the levo-lansoprazole response. This result indicates that the developed MIP sensor exhibited specific recognition for the target molecule. Since the polylysine–phenylalanine complex framework provided multiple recognition sites for the target molecules, the spatial conformation was better maintained, thereby improving the precision of the imprinted pores and reducing the interference from the other enantiomers during recognition.

### 3.7. Stability, Reproducibility, and Shelf Life

After five consecutive measurements of a levo-lansoprazole solution (1.0 × 10^−12^ mol/L) with the MIP sensor, a relative standard deviation (RSD) of 3.5%was obtained, reflecting the favorable stability of the sensor. Analyses of a levo-lansoprazole solution (1.0 × 10^−12^ mol/L) using five sensors prepared using the same method in the same batch gave an RSD of 3.9%, indicating good reproducibility. Sensors prepared in the same batch were stored in a refrigerator for five days and then used to analyze a levo-lansoprazole solution (1.0 × 10^−12^ mol/L) five consecutive times. No significant changes in the signal intensity were observed; however, after storage for 10 days, the signal intensity decreased by an average of 7.8%. These results indicate that the MIP sensor has a relatively long shelf life.

### 3.8. Anti-Interference Ability of the Sensor

**Figure 7:**
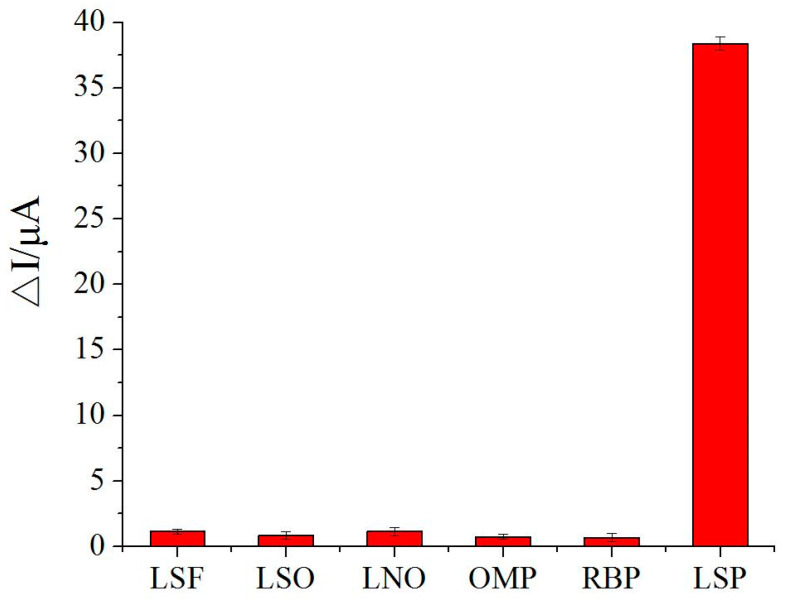
Anti-interference ability of the MIP sensor.

The anti-interference ability of the prepared MIP sensor was studied using substances with molecular structures similar to that of levo-lansoprazole. Rebinding experiments were performed using LSF, LSO, LNO, OMP, and RBP at a concentration of 1.0 × 10^−9^ mol/L. The recognition capabilities of the sensor for these interfering substances were compared with that for the target molecule levo-lansoprazole. As shown in Figure 7, the maximum response signal intensity among the interference substances was only 3.00% of the signal intensity for levo-lansoprazole, indicating that the sensor had low recognition ability for the interfering substances. These results clearly demonstrate that the MIP sensor exhibited specific recognition ability for the target molecule.

### 3.9. Real Sample Detection

Using a rebinding time of 10 min, the DPV response for the MIP sensor was measured for the sample solutions prepared using enteric-coated lansoprazole tablets and human serum samples. The accuracy of the sensor was assessed using the standard addition recovery method. The recovery rate of levo-lansoprazole in the sample was between 96.4% and 102.8% (Table 1), indicating the successful detection of levo-lansoprazole in enteric-coated lansoprazole tablets and in turn demonstrating the practical applicability of this sensor for the analysis of real samples.

## 4. Conclusions

In this study, a polylysine–phenylalanine complex was introduced during the preparation of the MIP sensors. The self-assembled framework of the polylysine–phenylalanine complex was employed to immobilize the target molecule, levo-lansoprazole, through hydrogen bonding. Owing to its highly ordered 3D structure, the polylysine–phenylalanine complex framework anchored the spatial conformation of the target molecule and improved the structural fineness of the imprinted pores, which increased the number of recognition sites and enhanced the specific recognition ability of the sensor. The excellent performance of the proposed MIP sensor in the analysis of real samples indicates its significant potential for practical applications. As the sensor is not restricted by the structure of the target molecule, this approach can serve as a novel platform for the separation and recognition of chiral drugs.

## Figures and Tables

**Table 1:** Recovery results for levo-lansoprazole in enteric-coated lansoprazole tablets and serum.

Samples	Found 10^−12^ mol/L	RSD% *n* = 5	Added 10^−12^ mol/L	Total Found 10^−12^ mol/L	RSD% *n* = 5	Recoveries %
Tablet 1	1.37	3.2	3.00	4.45	4.7	102.67
Tablet 2	1.72	3.5	3.00	4.78	4.4	102.00
Tablet 3	2.15	3.3	5.00	6.97	5.1	96.40
Tablet 4	1.94	3.1	5.00	7.08	4.9	102.80
Serum 1	ND	ND	3.00	2.98	4.2	97.80
Serum 2	ND	ND	5.00	5.10	3.8	101.26
Serum 3	ND	ND	10.00	9.89	4.6	100.79

ND: Not detected.

## Data Availability

Data is contained within the article or Supplementary Material.

## Supplementary Materials

The following supporting information can be downloaded at: https://www.mdpi.com/article/10.3390/bios13050509/s1: Figure S1: Effect of the polylysine-phenylalanine complex frame self-assembled time on the DPV intensity. Figure S2: Effect of the target molecular self-assembled time on the DPV intensity. Figure S3: Effect of the number of polymerization cycles on the DPV intensity. Figure S4: Effect of the elution time on the DPV intensity. Figure S5: Effect of the rebinding time on the DPV intensity. Figure S6: The EDS characterizations of bare gold electrode (A), nMIP film (B), MIP film (C) and MIP film after elution (D) Table S1: Performance comparison of different methods for the determination of levo-lansoprazole [24,25,26,27,28,29].
