# Supplementary material for: Development of Levo-Lansoprazole Chiral Molecularly Imprinted Polymer Sensor Based on the Polylysine–Phenylalanine Complex Framework Conformational Separation
Source: Biosensors (Basel). 2023 Apr 28;13(5):509. doi: 10.3390/bios13050509 (PMC10216006; doi:10.3390/bios13050509)
Supplement: Supplementary file 1 [file biosensors-13-00509-s001.zip › biosensors-2265977-supplementary.pdf]

Supplementary Material

Development of Levo-Lansoprazole Chiral Molecularly Imprinted Polymer Sensor Based on the Polylysine–Phenylalanine Complex Framework Conformational Separation

Lianming Zhang [1,*], Zian Wang [1], Dan Li [1], Yali Yuan [1,*], Huixiang Ouyang [2,*] and Jianping Li [1]

[1] College of Chemistry and Bioengineering, Guilin University of Technology, Guilin, 541004, China
[2] College of Chemistry & Environment Engineering, Baise University, Baise, 533000, China
* Correspondence: lianming226@126.com (L.Z.); thanksin2013@163.com (Y.Y.); huixiang73@163.com (H.O.); Tel.: +86-773-589-5622 (L.Z.)

1. Optimization of experimental conditions

The self-assembly time of the polylysine–phenylalanine-complex framework was optimised to explore its influence on the complex's incubation effect on the gold electrode surface. As shown in Fig.S1, the self-assembly time for the polylysine–phenylalanine-complex framework was set to 10.0, 15.0, 20.0, 25.0, 30.0, 35.0, and 40.0 min, and the corresponding response current intensities were measured. Experimental results showed that, when the self-assembly time exceeded 30 min, the response current intensity tended to be constant, indicating that the polylysine–phenylalanine-complex framework had stably self-assembled on the electrode surface. Therefore, 30.0 min was selected as the optimal self-assembly time for the polylysine–phenylalanine-complex framework in subsequent experiments.

The target molecule, levo-lansoprazole (1.0×10^{-4} mol/L), was self-assembled on the electrode modified with the polylysine–phenylalanine-complex framework. The self-assembly time was set to 10.0, 15.0, 20.0, 25.0, 30.0, and 35.0 min, respectively, and the corresponding response current intensities were measured. As shown in Fig.S2, the response signal intensity of the self-assembled target molecule, levo-lansoprazole, decreased with time. This experiment found that when the self-assembly time exceeded 25.0 min, the response signal intensity of the probe molecule became relatively constant; that is, 25.0 min was the optimal self-assembly time for the target molecule. Therefore, 25 min was selected as the self-assembly time for the target molecule in subsequent experiments.

The number of polymerization cycles was studied to understand its influence on the MIP membrane stability and the imprinting effect. The electropolymerisation of *o*-phenylenediamine (1.0×10^{-4} mol/L) on the gold electrode surface is an irreversible process. As shown in Fig.S3, near –0.46 V, the characteristic oxidation peak of *o*-phenylenediamine appeared and the oxidation peak current was the largest during the first cycle of electropolymerisation. With an increase in the number of polymerization cycles, the response signal intensity decreased, and the electrode surface was uniformly covered by an *o*-phenylenediamine polymer membrane with poor conductivity. By comprehensively considering the stability of the polymer membrane, the elution effect, and the re-adsorption recognition effect, the optimal result was observed upon reaching eight polymerization cycles. Therefore, eight was selected as the optimal number of cycles for polymerization in subsequent experiments.

The elution effects of different eluents on the target molecule, levo-lansoprazole, were studied here. Specifically, ethanol, acetic acid, ethanol: H_2O mixture (2/8, *V*/*V*), acetic acid: H_2O mixture (2/8, *V*/*V*), and ethanol: acetic acid: H_2O mixture (2/3/8, *V*/*V*/*V*) were separately adopted as the eluent to elute levo-lansoprazole. By comprehensively considering the elution effect, elution time, stability of the imprinted membrane, and stability of the polylysine-phenylalanine-complex framework, the best elution effect was achieved when using the ethanol: acetic acid: H_2O mixture (2/3/8, *V*/*V*/*V*). Subsequently, with the ethanol: acetic acid: H_2O mixture (2/3/8, *V*/*V*/*V*) as the eluent, the response signal intensi-

ties were tested using elution times of 1.0, 2.0, 3.0, 4.0, 5.0, 6.0, 7.0, 10.0, and 15.0 min, respectively. As shown in Fig.S4, the reduction peak current intensity of the probe molecule after elution increased with an increase in the elution time. Experiments showed that the elution time of 5.0 min delivered the best elution effect. After the elution time exceeded 5.0 min, the elution response signal intensity basically remained unchanged. Therefore, 5.0 min was selected as the optimal elution time in subsequent experiments.

To further optimize the experiment and avoid excessive time consumption, the rebinding time was optimized. The prepared sensor was immersed in the levo-lansoprazole solution with a concentration of 1.0×10^{-12} mol/L for rebind, and the rebinding time was set to 5.0, 6.0, 7.0, 8.0, 9.0, 10.0, 11.0, 12.0, 15.0, and 20.0 min, respectively. Experiments in Fig.S5 showed that when the rebinding time exceeded 10.0 min, the response current intensity of the probe molecule decreased gradually and finally tended to be flat. Therefore, 10.0 min was selected as the re-adsorption time in subsequent experiments.

2. EDS characterizations

The elements of different coatings were characterized by EDS (Fig.S6), including bare gold electrode (A), nMIP membrane (B), MIP membrane (C) and eluted MIP membrane (D). As shown in the figure, when there is no other modification on the surface of the bare gold electrode, the elemental characterization is only Au. When the membranes of nMIP, MIP and eluted MIP were modified, the proportion of elements changed. Compared with MIP membrane, nMIP membrane does not contain characteristic elements S and F from levo-lansoprazole, but only C, N and O from *o*-phenylenediamine, which is attributed to the absence of levo-lansoprazole in the preparation of nMIP membrane. On the contrary, the S (4.18%) and F (0.21%) elements of levo-lansoprazole appeared in the MIP prepared by adding levo-lansoprazole. The proportion of S and F in the eluted MIP membrane decreased to 2.8% and 0.11%, respectively, indicating that levo-lansoprazole in the MIP membrane was successfully removed.

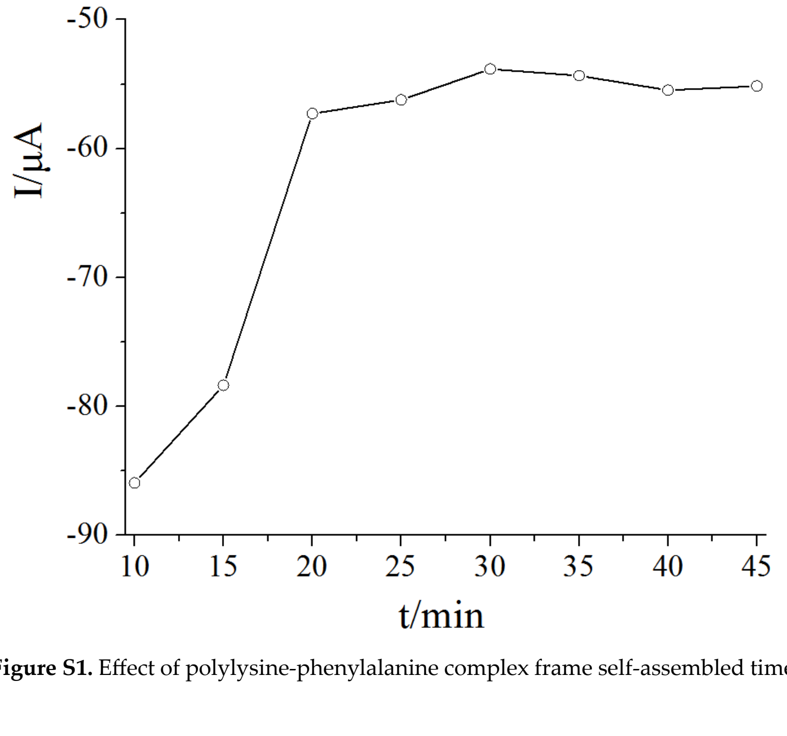

Figure S1. Effect of polylysine-phenylalanine complex frame self-assembled time on DPV intensity.

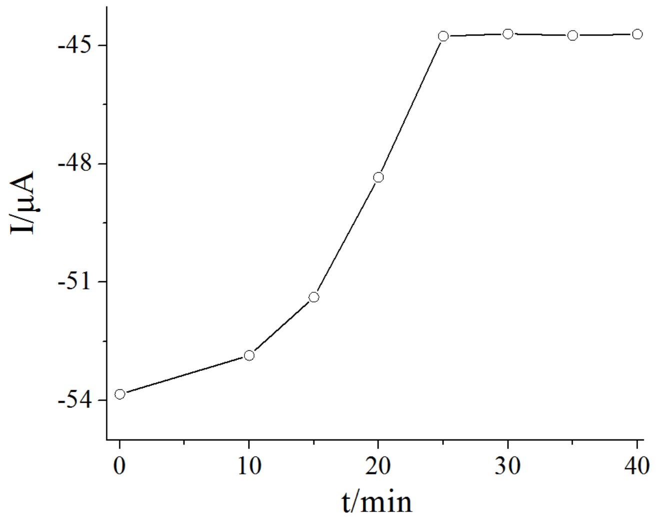

Figure S2. Effect of target molecular self-assembled time on DPV intensity.

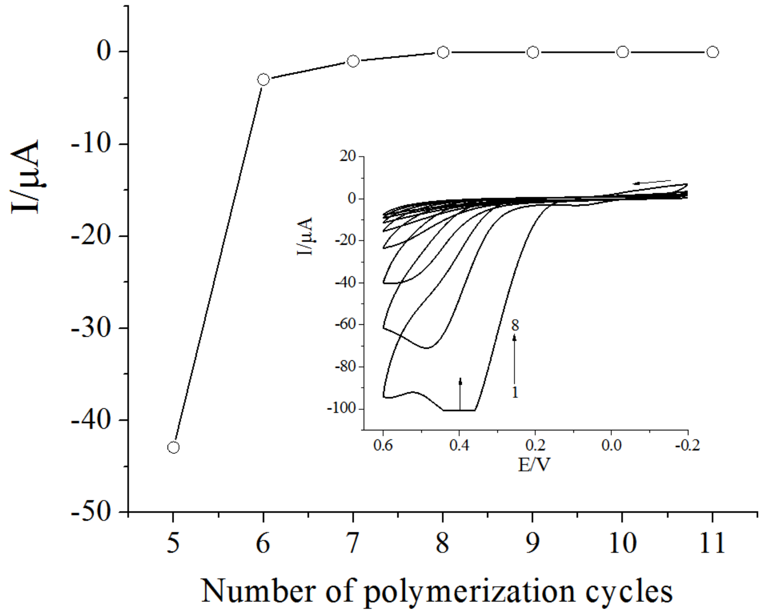

Figure S3. Effect of number of polymerization cycles on DPV intensity.

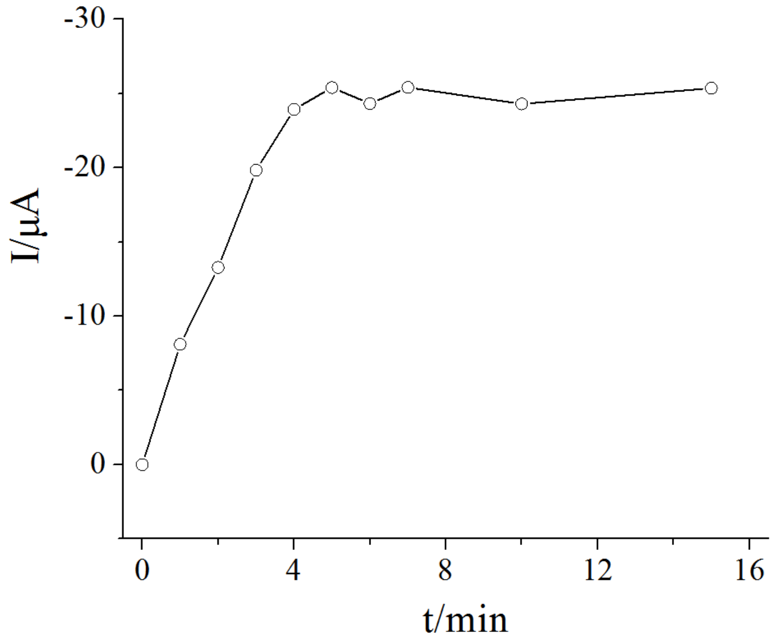

Figure S4. Effect of elution time on DPV intensity.

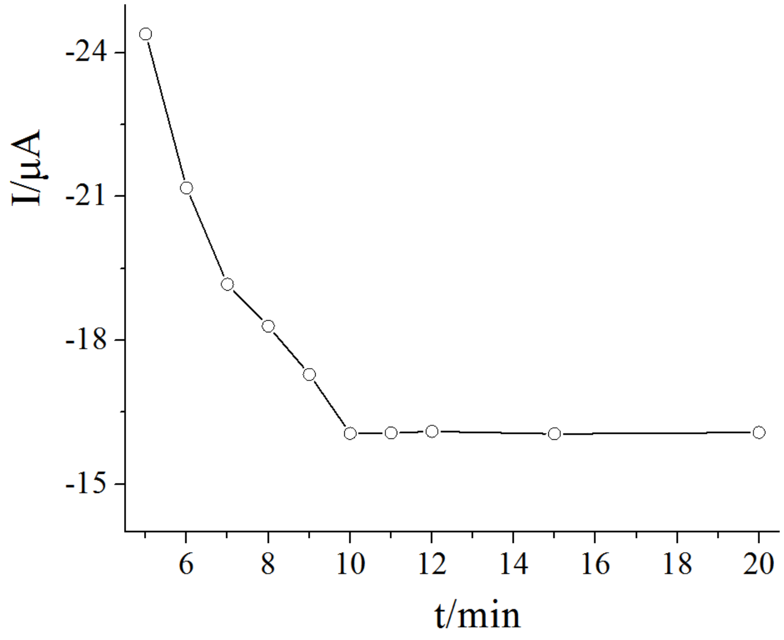

Figure S5. Effect of rebinding ime on DPV intensity.

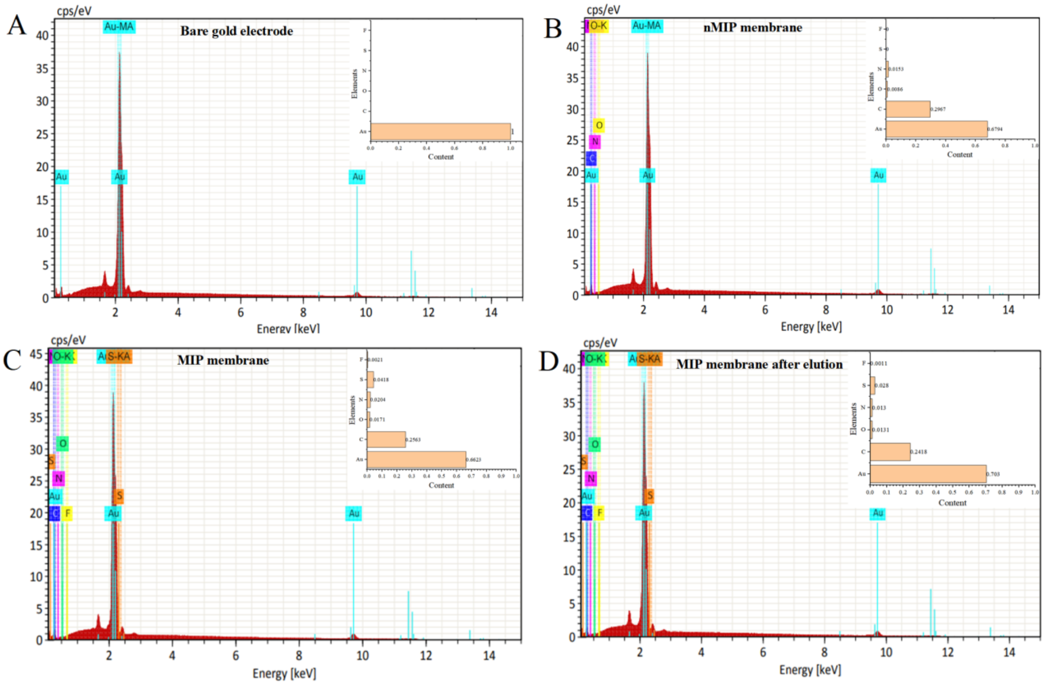

Figure S6. The EDS characterizations of bare gold electrode (A), nMIP film (B), MIP film (C) and MIP film after elution (D).

Table S1. Performance comparison of different methods for determination of levo-lansoprazole.

Assay	Linear range (mol/L)	LOD (mol/L)	Analysis time (min)	Reference
HPLC-UV–Vis	$6.77 \times 10^{-6} \sim 4.06 \times 10^{-5}$	6.50×10^{-7}	4.5	[1]
MIP-PGE	$1.00 \times 10^{-5} \sim 1.00 \times 10^{-3}$	3.10×10^{-8}	——	[2]
Capillary electrophoresis	$1.12 \times 10^{-5} \sim 2.24 \times 10^{-4}$	1.40×10^{-6}	——	[3]
FI-CL	$2.71 \times 10^{-8} \sim 2.71 \times 10^{-5}$	5.41×10^{-9}	——	[4]
PVC membrane	$1.00 \times 10^{-6} \sim 1.0 \times 10^{-2}$	1.57×10^{-5}	≤ 0.5	[5]
Chemical nanosensor	$2.17 \times 10^{-6} \sim 2.38 \times 10^{-5}$	2.52×10^{-7}	40	[6]
MIP/polylysine–phenylalanine complex framework	$1.0 \times 10^{-13} \sim 3.0 \times 10^{-11}$	3.0×10^{-14}	10	This work

——：not found.

1. Gülfen, M.; Canbaz, Y.; Özdemir, A. Simultaneous determination of amoxicillin, lansoprazole, and levofloxacin in pharmaceuticals by HPLC with UV–Vis detector. *J. Anal. Test*. **2020**, 4, 45-53.
2. Khalili, Z.; Nezhadali, A. Nanomolar detection of lansoprazole: computational–assisted to monomer–templet complex study based on molecularly imprinted polymer and electrochemical determination. *Chem. Pap*. **2022**, 76, 1185–1198.
3. Dogrukol-Ak, D.; Tuncel, M.; Aboul-Enein, H.Y. The determination of lansoprazole in pharmaceutical preparation by capillary electrophoresis. *Chromatographia* **2021**, 54, 527-530.
4. Khan, M.S.; Asghar, M.; Yaqoob, M.; Ali, S.; Haider Shah, S.; Siddiqui, M.A. Determination of lansoprazole in pharmaceuticals using flow injection with rhodamine 6G–diperiodatoargentate (III) chemiluminescence detection. *Luminescence*, **2022**, 37(7), 1126-1134.
5. Rahman, N.; Khan, S. Experimental design approach in the optimization of potentiometric method for lansoprazole determination using lansoprazole-tungstate based ion-selective electrode. *Ind. Eng. Chem. Res*3**2018**, 57(29), 9351-9361.
6. Nassar, M.Y.; El-Moety, E.A.; El-Shahat, M.F. Synthesis and characterization of a $ZnMn_2O_4$ nanostructure as a chemical nanosensor: a facile and new approach for colorimetric determination of omeprazole and lansoprazole drugs. *RSC adv*. **2017**, 7(69), 43798-43811.
